# Syringic Acid Attenuates Cardiomyopathy in Streptozotocin-Induced Diabetic Rats

**DOI:** 10.1155/2021/5018092

**Published:** 2021-12-28

**Authors:** Zahra Sabahi, Mohammad Javad Khoshnoud, Sara Hosseini, Fatemeh Khoshraftar, Marzieh Rashedinia

**Affiliations:** ^1^Medicinal Plants Processing Research Center, Shiraz University of Medical Sciences, Shiraz, Iran; ^2^Food and Supplements Research Center, Shiraz University of Medical Sciences, Shiraz, Iran; ^3^Department of Pharmacology and Toxicology, School of Pharmacy, Shiraz University of Medical Sciences, Shiraz, Iran

## Abstract

**Objectives:**

Diabetic cardiomyopathy (DC) has become one of the serious complications in diabetic cases. In this study, we aimed to explore the syringic acid (SYR) protective effect against diabetes-induced cardiac injury in experimental rats.

**Methods:**

Rats were divided in control and streptozotocin-induced diabetic rats which were subdivided into diabetic controls, and three test groups (SYR at 25, 50, and 100 mg/kg) and the nondiabetic group received 100 mg/kg of SYR. All treatments were given SYR for 6 weeks. SYR effects on cardiac diagnostic markers, heart lipid peroxidation, protein carbonylation, antioxidant system, and changes of the heart mitochondrial mass and biogenesis were measured.

**Results:**

Diabetes induction prompted CK-MB, LDH levels in serum, cardiac catalase, and superoxide dismutase activity, as well as cardiac TBARs and carbonylated protein. SYR administration (100 m/kg) attenuated CK-MB and LDH levels. Also, 50 and 100 mg/kg of SYR reduced cardiac TBARs and carbonylated protein in diabetic rats. These treatments did not show any effects on GSH content, mtDNA, and mitochondrial biogenesis indices (PGC1- *α*, NRF1, NRF2, and TFAM) in heart tissue.

**Conclusions:**

SYR treatment showed protective effects on diabetic cardiomyopathy in rats by reducing lipid peroxidation and protein carbonylation. The possible mechanisms could be related to antioxidant activity of this phenolic acid. SYR might play a role of a protective factor in cardiac challenges in diabetes.

## 1. Introduction

Diabetic cardiomyopathy (DC) is one of the serious complications in diabetic patients. It is known by increasing some biomarkers such as low-density lipoprotein, glucose, glycated hemoglobin levels, and fibrotic markers insulin-like growth factor and transforming growth factor levels, accompanied by severe diastolic dysfunction. Among other diabetic complications, DC is responsible for almost 80% of mortality in patients suffering from diabetes [1].

Hyperglycemia, insulin resistance, and increased fatty acid metabolism are associated with DC progress. Also, some alterations in the cardiomyocytes are responsible for DCM. These alterations comprise apoptosis, diastolic dysfunction, inflammation, and imbalanced calcium homeostasis [1, 2].

Mitochondria are the main source of ATP in the heart. As the heart is known as the high-metabolism-demanding organ, mitochondrial dysfunction is considerable in this organ [1].

It seems that mitochondrial dysfunction is associated with metabolic disorders and insulin resistance-related heart disease. Hence, uncontrolled chronic hyperglycemia and insulin resistance in diabetic condition give rise to alter cardiac mitochondrial dynamics and function [2].

In this complication, mitochondrial dysfunction leads to overproduction of cellular reactive nitrogen species (RNS) and reactive oxygen species (ROS) levels [3].

High level of ROS in the diabetic myocardium can be the cause of molecular and cellular myocardial damages and irregular morphology and function. On the other hand, in the diabetic heart, reduction of antioxidant defences and excessive oxidants lead to oxidative stress which make cells prone to oxidative damages [1].

Moreover, cardiac diastolic is affected by high level of advanced glycated end products in the heart. A previous report showed that oxidative stress is able to change contractile proteins which play a critical role in the progress of heart failure [3].

Nowadays, there is a great consideration to using phytochemicals in order to reduce diabetes complications and oxidative stress-related disease. Studies have indicated that natural antioxidants such as polyphenols were effective in controlling these complications [4–7]. Some studies exhibited the cardioprotective role of phenolic acids. Yogeeta et al. recommended ferulic acid as a protective factor in the antioxidant defence system during induced myocardial infarction. Also, this compound could restore the cardiac mitochondrial function of induced myocardial infarction in rats [8]. In another study, salvianolic acids showed cardioprotection ability against adriamycin-induced toxicity by reducing oxidative stress [9]. Kumaran et al. also reported that caffeic acid protects cardiac mitochondrial function and structure in isoproterenol-induced oxidative damages [10]. This phenolic acid is an efficient superoxide radical scavenger which is formed in the mitochondria [11].

Syringic acid (SYR), a derivative of hydroxybenzoic acid, is a plant secondary metabolite and known as a phenolic acid. This phenolic compound is famous antioxidant, and the presence of methoxy groups attached to the phenyl ring of the SYR structure helps it to play a role as a free-radical scavenging factor [5, 12]. SYR is considered to be an anti-inflammatory, antidiabetic agent [13, 14]. Previous reports showed that that SYR was able to improve hepatic [12], nephropathy [15], and neuropathy [16] complications in diabetic rats.

As oxidative stress plays a critical role in diabetic complications, it seems that natural antioxidants would be efficient in these complications. The aim of this study was to determine the effects of SYR on oxidative stress and mitochondrial biogenesis in the heart of streptozotocin-induced diabetic rats.

## 2. Materials and Methods

### 2.1. Chemicals

All chemicals of this experiment were purchased from Sigma-Aldrich (St. Louis, USA) or Merck Company (Darmstadt, Germany), the highest grade commercially available.

### 2.2. Animal Model

The Sprague Dawley rats (weight 220–240 g) were used in this study obtained from the Center of Comparative and Experimental Medicine of Shiraz University of Medical Sciences (Shiraz, Iran). They were housed under standard conditions and treated with a standard rat diet and water *ad libitum* (22 ± 2°C and 12 h light/dark cycle).

The experiment was performed according to the procedures approved by the local ethics committee (Shiraz University of Medical Sciences, Shiraz, Iran, permit # IR.SUMS.REC.1395.S969) in agreement with the Guide for the Care and Use of Laboratory Animals.

To induce diabetes, rats were treated with a single i.p. dose of STZ (60 mg/kg). Diabetes was confirmed 7 days after the STZ injection, by measuring blood glucose level. Blood samples were taken from the tail vein, and glucose level was evaluated by using a glucometer (Gluco Lab, Korea). Rats with fasting blood glucose levels more than 300 mg/dl were used in this study [17].

### 2.3. Study Design

The experimental design consists of 6 groups of rats; each group contains 6 rats: nondiabetic control, diabetic, diabetic + SYR (25, 50, and 100 mg/kg), and nondiabetic SYR (100 mg/kg). SYR was administered daily via intragastric gavage for six weeks. Only higher dose of SYR (100 mg/kg) was performed to prove the nontoxic effects of SYR on nondiabetic rats.

The rats were treated with SYR using an intragastric tube daily for a period of 6 weeks. After depriving of food for 12 h, all the rats were anesthetized by high dose of pentobarbital sodium (60 mg/kg bodyweight) and sacrificed by cervical decapitation. Then, the blood samples were collected in a tube for analysis of serum biochemical markers. The hearts were removed and washed in ice-cold saline, and separated tissue fragments were weighted and homogenized in cold phosphate buffer (PBS, 0.1 M, pH = 7.4) for biochemical analysis and RNA and DNA extraction. Homogeneous tissues were aliquoted and then frozen at −70°C until use.

### 2.4. Assay of Cardiac Diagnostic Serum Markers

Level of serum creatinine kinase-MB (CK-MB) and lactate dehydrogenase (LDH) activities were assayed by using commercial kits purchased from Pars Azmoon (Iran) according to the manufacturer's instructions.

### 2.5. Measurement of TBARs, GSH, SOD, and CAT in Heart Tissue

Lipid oxidation of cardiac tissue was determined by evaluating the malondialdehyde (MDA) level.

In this assay, MDA reacts with thiobarbituric acid forming chromophore (TBARs), which is measured at 535 nm [15, 18].

We reduced the glutathione (GSH) content of tissue reacts with Ellman's reagent (DTNB) to form the yellow-colored compound which is measured at 412 nm [19], and the catalase activity (CAT) was measured by means of the previous description [20, 21]. Superoxide dismutase (SOD) activity was measured through a commercial kit (Nasdox, Navand salamat, Iran) [22].

### 2.6. Nitric Oxide Measurement

In brief, Griess reagents, sulfanilamide, and N-(1-naphthyl) ethylendiamine dihydrochloride were added to the supernatant of homogenated cardiac tissues and incubated at 37°C for 30 min; then, absorbance was read at 540 nm [23].

### 2.7. Evaluation of Carbonyl Protein

The Kiazist Protein Carbonyl Colorimetric Assay Kit (Iran) was used for the measurement of oxidized proteins. The protocol is based on derivatization by use of the reaction between 2,4-dinitrophenylhydrazine and protein carbonyls. Total protein concentrations in all homogenated tissue were measured using the Bradford method and normalized [24].

### 2.8. Quantitative Real-Time PCR

Total RNA was extracted from cardiac tissues by using Trizol reagent (Pars Tous, Mashhad, Iran), and then, RNA was converted to cDNA based on the manufacturer's protocol (Easy cDNA Synthesis Kit; Pars Tous, Iran). Real-time PCR was performed using a thermocycler and SYBR green detection system (Applied Biosystems, USA). Primer sequences were: 18S: 5ʹ-CGAACGTCTGCCCTATCAACTT-3ʹ, 5ʹ-CTTGGATGTGGTAGCCGTTTCT-3ʹ;PGC-1*α*: 5ʹ-CGGGATGGCAACTTCAGTAAT-3ʹ, 5ʹ-AAGAGCAAGAAGGCGACACA-3ʹ;NRF1: 5ʹ-GGGGAACAGAACAGGAAACA-3ʹ, 5ʹ-CCGTAATGCACGGCTAAGTT-3ʹ;NRF2: 5ʹ-GGGGAACAGAACAGGAAACA-3ʹ, 5ʹ-CCGTAATGCACGGCTAAGTT-3ʹ; and TFAM: 5ʹ-GAAAGCACAAATCAAGAGGAG-3ʹ, 5ʹ CTGCTTTTCATCATGAGACAG-3ʹ. The mRNA expression levels were indicated with 2-ΔΔCT and normalized to 18s rRNA [25].

### 2.9. Determination of mtDNA Copy Number

Total genomic DNA was achieved from cardiac tissue by using a commercial kit (Pars Tous, Iran). The mitochondria DNA copy was measured and mtDNA (RNR2)/nDNA (GAPDH) using real-time PCR. Primer sequences were nuclear-encoded GAPDH: 5′-GGAAAGACAGGTGTTTTGCA-3′, 5′-AGGCAGAGTGAGCAGGACA-3′; RNR2:5′-AGCTATTAATGGTTCGTTTGT-3′, 5′-AGGAGGCTCCATTTCTCTTGT-3′.

### 2.10. Statistical Analysis

Data are expressed as mean ± SEM. The significance among different groups was calculated by using one-way ANOVA followed by Dunnett's test. GraphPad Prism 6.0 software was used for analysis and plotting graphs. Differences were considered statistically significant for *P* values < 0.05.

## 3. Results

### 3.1. The Effect of SYR on Biochemical Indexes in Serum and Tissue

**Figure 1:**
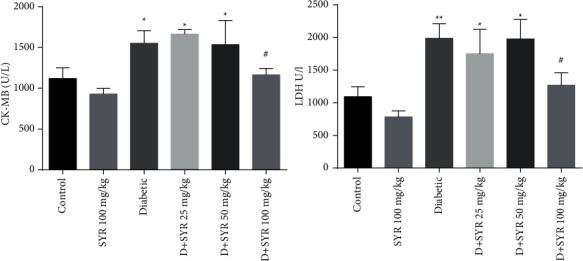
Effect of syringic acid on heart injury markers (serum CK-MB and LDH levels) of STZ-induced diabetic rats in various experimental groups. SYR: syringic acid; *D* + SYR: diabetic + syringic acid. All values are expressed as mean ± S.E.M.; *n* = 6 per group. ^*∗*^*P* < 0.05 and ^*∗∗*^*P* < 0.01 vs. the control group; ^#^*P* < 0.05 vs. the diabetic group.

According to our results, serum CK-MB and LDH levels (Figures 1(a) and 1(b)) increased significantly in the diabetic group as compared to the control rats (*P* < 0.05, *P* < 0.01, respectively). Administration of SYR (25 and 50 mg/kg) could not reduce these factors in diabetic rats. However, SYR (100 mg/kg) reduced CK-MB and LDH levels in the diabetic treated group (*P* < 0.05). Also, administration of 100 mg/kg of SYR did not change these markers in nondiabetic control rats.

**Figure 2:**
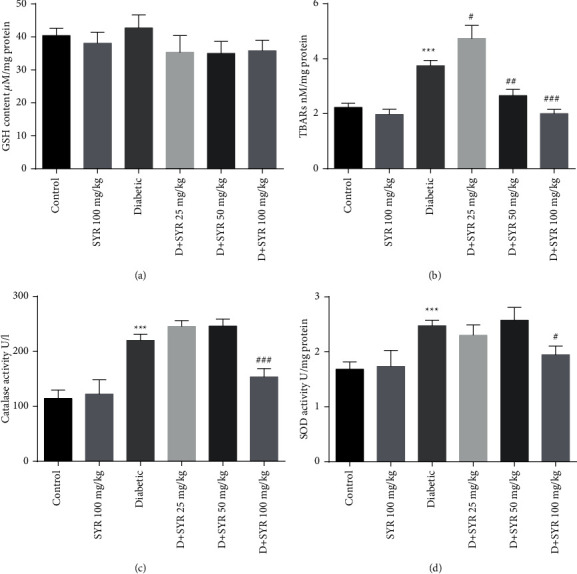
Effect of syringic acid on oxidative stress and antioxidant markers in the heart of STZ-induced diabetic rats in various experimental groups. GSH: reduced glutathione (a); TBARS : thiobarbituric acid reactive substances (b); CAT: catalase (c); and SOD: superoxide dismutase (d). SYR: syringic acid; *D* + SYR: diabetic + syringic acid. All values are expressed as mean ± S.E.M.; *n* = 6 per group. ^*∗*^*P* < 0.05 and ^*∗∗∗*^*P* < 0.01 vs. the control group; ^#^*P* < 0.05, ^##^*P* < 0.01, and ^###^*P* < 0.001 vs. the diabetic group.

The GSH content of the heart did not show any significant changes in diabetic control and treated groups (Figure 2(a)).

The rats induced with STZ showed a significant increase (*P* < 0.001) in the levels of TBARs in the heart tissue when compared to the normal control. Treatment of diabetic rats with SYR (50 and 100 mg/kg) significantly decreased (*P* < 0.01 and *P* < 0.001, respectively) the levels of TBARs in the heart of diabetic rats (Figure 2(b)).

Elevated activity of cardiac CAT and SOD activities were determined in untreated diabetic rats as compared with control (*P* < 0.001). Treatment of diabetic rats with SYR (25 and 50 mg/kg) for 6 weeks did not show significant effects on cardiac CAT and SOD activities as compared to the diabetic group. Treatment of diabetic rats with SYR (100 mg/kg) significantly reduced the activities of CAT and SOD in them (*P* < 0.001 and *P* < 0.05, respectively) (Figures 2(c) and 2(d)).

**Figure 3:**
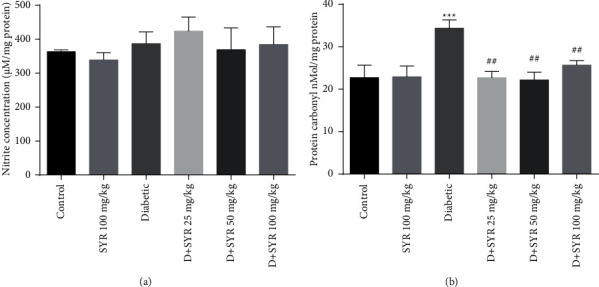
The results of nitrite content (a) and the levels of protein carbonyl (b) in diabetic rats in the heart tissue. SYR: syringic acid; *D* + SYR: diabetic + syringic acid. All values are expressed as mean ± S.E.M.; *n* = 6 per group. ^*∗∗∗*^*P* < 0.01 vs. the control group; ^##^*P* < 0.01 and ^###^*P* < 0.001 vs. the diabetic group.

The results of measuring nitrite content in the heart tissue are presented in (Figure 3(a)). No significant differences were observed between control and diabetic groups or diabetic and SYR-treated groups.

The levels of protein carbonyl in the diabetic group significantly increased when compared to control (*P* < 0.001). Treatment of diabetic rats with SYR (25, 50, and 100 mg/kg) reduced the protein carbonyl content levels of that groups significantly (*P* < 0.01) (Figure 3(b)).

### 3.2. The Effect of SYR on Mitochondrial Biogenesis and Mitochondrial Mass

**Figure 4:**
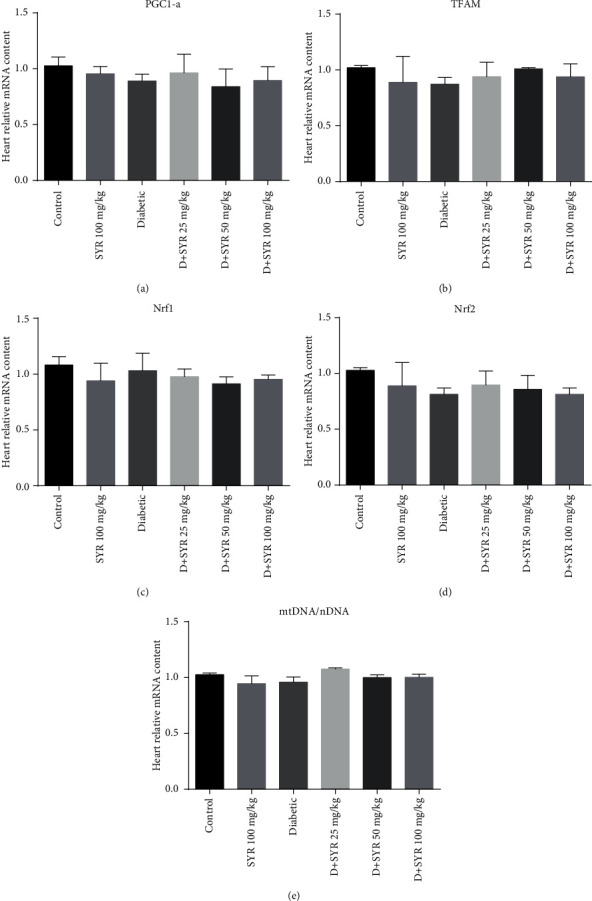
Effect of syringic acid on mitochondrial biogenesis factors in the heart of STZ-induced diabetic rats in various experimental groups. The mRNA expression of mitochondrial biogenesis factors was measured by real-time RT-PCR; PGC1-*α*; NRF1; NRF2; and TFAM. The relative mitochondrial DNA content was quantified: mtDNA/nDNA. SYR: syringic acid; *D* + SYR: diabetic + syringic acid. All values are expressed as mean ± SEM.; *n* = 3 per group.

The mRNA expression levels of PCG-1*α*, NRF-1, NRF-2, and TFAM in the heart were analysed, since they are markers of the mitochondrial biogenesis. The mRNA levels of PGC-1*α*, NRF-1, NRF-2, and TFAM were not changed in the diabetic group in comparison with the control group. Administration of SYR in diabetic rats was not effective in the alteration of the mRNA levels of PGC-1*α*, NRF-1, NRF-2, and TFAM. Also, there was no significant difference between mtDNA/nDNA content in the heart of the control, diabetic control, and diabetic treated group after treatment with SYR (Figure 4).

## 4. Discussion

The aim of this study was to reveal the potential therapeutic effects of SYR on diabetes cardiac complications. Based on our previous research, SYR played protective roles in the liver, kidney, and brain of diabetic rats [12, 15, 16]. Based on previous reports, three doses comprising 25, 50, and 100 mg/kg of SYR were selected as safe and effective doses in diabetes. In these reports, it was seen that 100 mg/kg of SYR was able to reduce hyperglycemia in diabetic rats [12, 15]. Different mechanisms such as increasing insulin level, restoring insulin sensitivity, and improving the consumption of glucose by peripheral tissues are responsible for SYR antihyperglycemic effects [26].

Leakage of CK-MB and LDH is the result of cell membrane damages and elevated membrane permeability. LDH and CK-MB are known as significant biomarkers for cardiac injury [27]. In this study, it was found that diabetes induction causes an increase in the amount of diagnostic marker enzyme, CK-MB and LDH; treatment with SYR (100 mg/kg) decreased the elevated level of CK-MB.

In previous studies, treatment of diabetic rats with gallic acid significantly improved alterations of CK-MB and LDH in the diabetic group [27]. Also, pretreatment of p-coumaric acid in isoproterenol-induced myocardial infarcted rats reduced the activity/level of these cardiac markers [28]. There is a strong relationship between diabetes and oxidative stress. Constant hyperglycemia is the cause of this cellular stress, and the presence of excessive ROS can damage cellular proteins, lipids, and nucleic acids. The lipid peroxidation products (TBARS) were increased in diabetic rats.

Elevated level of lipid peroxidation in the diabetic group was in agreement with the results of other studies in the heart [29, 30]. Hyperglycemia induces oxidative reactions in lipids, and myocardial membrane damages lead to cardiac lipid peroxidation. Also, lipid accumulation in the heart makes this organ sensitive to lipid peroxidation [27]. Treatment of diabetic rats with SYR (50 and 100 mg/kg) reduced the elevated level of TBARS. A previous report showed that caffeic acid and ellagic acid could alleviate cardiac oxidative stress by reducing the MDA formation in cardiac tissue of diabetic mice [31].

In the current study, elevated activity of cardiac CAT and SOD activities were determined in untreated diabetic rats. These results were similar to the results of other studies [32–35]. Conversely, some other reports revealed decreased CAT and SOD activities [36–38].

As antioxidant enzyme activities are associated with ROS concentration, the possible reason of the current results is that, during the six weeks, ROS production attributable to diabetes was just slightly increased and had stimulated the rise of CAT and SOD activities [32].

The increased catalase activity in the heart and vessels could be a compensatory process to reduce oxidative stress, particularly, peroxidative stress and high level of hydrogen peroxide (H_2_O_2_) in vessels [33]. Catalase is able to detoxify high concentrations of H_2_O_2_ while GSHPx is effective at lower concentrations of H_2_O_2_. So, in a high H_2_O_2_ level, induction of catalase in diabetic vessels is expected.

Increased level of CAT may also be related to the location of this enzyme. The catalase and fatty acyl CoA oxidase both are located in the peroxisomal compartment. Fatty acyl CoA oxidase is responsible of H_2_O_2_ generation by fatty acid and keton body which is increased in diabetes state [33].

Treatment of diabetic rats with SYR (100 mg/kg) significantly reduced the activities of CAT and SOD in them.

As SYR is an antioxidant, it is expected that SYR treatment reduces ROS production and subsequently decreases the activities of these enzymes. Similar results were obtained by others. For instance, the administration of folic acid decreases SOD and CAT activities in the heart of both healthy and diabetic rats [32], and, in other study, supplementation of olive oil polyphenols decreased SOD activity in human and rat plasma [39, 40] and chicken blood [41]. It could be suggested that antioxidants reduce SOD activity by scavenging superoxide anion directly as a compensation mechanism [41].

Although hyperglycemia induces ROS and reactive nitrogen species (RNS) production which are the major related factors in the development of diabetic cardiomyopathy [38], in our study, nitrite content in the heart tissue did not show any significant differences among control and diabetic groups or diabetic and SYR-treated groups.

Accumulation of protein carbonylation is a remarkable indicator for protein oxidation in some diseases, particularly in diabetes [42]. In the present study, diabetic rats exhibited a significantly elevated level of carbonylated protein. Treatment of diabetic rats with SYR (25, 50, and 100 mg/kg) reduced the elevated level of protein carbonylation in the heart significantly. In line with our study, protocatechuic acid, an isolated phenolic from *Sansevieria roxburghiana* leaves (50 and 100 mg/kg), could significantly alleviate protein carbonylation in the myocardial tissues of diabetic rats. Excess cellular ROS promotes lipid peroxidation and protein carbonylation, as well as reduction of antioxidant enzymes occur in the myocardial tissues of diabetic rats [43]. Consequently, SYR treatment was able to play a role of an ROS scavenger in the myocardial tissues following protection of lipids and proteins against oxidative damages. According to previous research, there is a relationship between impaired mitochondrial biogenesis and cardiac dysfunction in diabetes [44]. In the current study, the mRNA levels of PGC-1*α*, NRF-1, NRF-2, and TFAM were not changed in the heart of the diabetic group when compared with the control. Although PGC-1*α*, a transcriptional coactivator, has a noticeable role in the regulation of myocardial metabolism, there are controversial results reported in terms of PGC-1*α* expression in diabetes. On the other hand, either upregulated or unchanged in animal models of diabetes were reported which were associated with the age and the stage of diabetes in mice [45–47].

According to previous reports, NO is a possible signalling molecule, in the mitochondrial biogenesis process [48]. Also, oxygen or nitrogen reactive species are regulators of mitochondrial biogenesis in cardiac muscle, skeletal muscle, and adipose tissue [47]. In this study, we could not observe any change in NO content; consequently, NO was unable to play its role in the regulation of mitochondrial biogenesis. Also, it can be proposed that cardiac complication in diabetic groups in 6 weeks was not so intensive and they recovered by compensating the mechanism.

## 5. Conclusions

In conclusion, this study shows that syringic acid attenuates diabetic cardiomyopathy via amelioration of CK-MB and LDH. It reduces cardiac lipid peroxidation and protein carbonylation. It seems that syringic acid has protective potentials and combats these diabetic challenges. Also, these outcomes would encourage future studies to reveal the molecular mechanism of natural products in diabetic cardiomyopathy.

## Data Availability

The data used to support the findings of this study are included within the article.
